# Supplementary material for: Comparative Analysis of Epicuticular Lipids in Locusta migratoria and Calliptamus italicus: A Possible Role in Susceptibility to Entomopathogenic Fungi
Source: Insects. 2022 Aug 16;13(8):736. doi: 10.3390/insects13080736 (PMC9409248; doi:10.3390/insects13080736)
Supplement: Supplementary file 1 [file insects-13-00736-s001.zip › insects-1814066-supplementary.pdf]

ELECTRONIC SUPPLEMENTARY MATERIAL

Comparative analysis of epicuticular lipids in *Locusta migratoria* and *Calliptamus italicus*: a possible role in susceptibility to entomopathogenic fungi

Mariya D. Ganina[1], Maksim V. Tyurin[2], Ulzhalgas T. Zhumatayeva[3], Georgy R. Lednev[4], Sergey V. Morozov[1], Vadim Yu. Kryukov[2]

[1]N.N. Vorozhtsov Novosibirsk Institute of Organic Chemistry, Siberian Branch of Russian Academy of Sciences, Academician Lavrentyev Ave. 9, Novosibirsk 630090, Russia.
[2]Institute of Systematics and Ecology of Animals, Siberian Branch of Russian Academy of Sciences, Frunze Str. 11, Novosibirsk 630091, Russia
[3]Department of Plant Protection and Quarantine, Faculty of Agrabiology, Kazakh National Agrarian Research University, Abai Avenue 8, Almaty 050010, Kazakhstan
[4]All-Russian Institute of Plant Protection, Podbelskogo Avenue 3, St. Petersburg, Pushkin 196608, Russia

Text S1. The description of linear retention indices calculation, detection of characteristic ions in mass spectra and the identification of *n*-alkanes.

GC-MS. Linear retention indices (LRI) were calculated by means of retention times of *n*-alkanes C25–C40 from paraffin during a linear temperature increase. To detect characteristic ions in minor or poorly resolved peaks, the 3σ criterion was used. The analysis was based on a mass spectrum of a linear alkane. In the region *m/z* 120–350, ion abundance was normalized to the most abundant ion in the mass spectrum (*m/z* 57). Then, anamorphosis and linear regression were constructed. Interval ±3σ was built on the basis of the regression obtained along the *y*-axis (σ: standard deviation). In the mass spectra of methyl-branched alkanes, signals of putative characteristic ions were also normalized and linearized. If the abundance of an ion was above the upper limit of the 3σ interval, then this ion was assumed to be characteristic. Otherwise, the ion was not assumed to be characteristic.

Linear alkanes in the epicuticular extracts were identified via a comparison with retention times of *n*-alkanes from a paraffin blend as well as on the basis of their mass spectra: the absence of prominent characteristic ions corresponding to decay at branch points, a prominent molecular ion, and/or the absence or very low abundance of ion M-15.

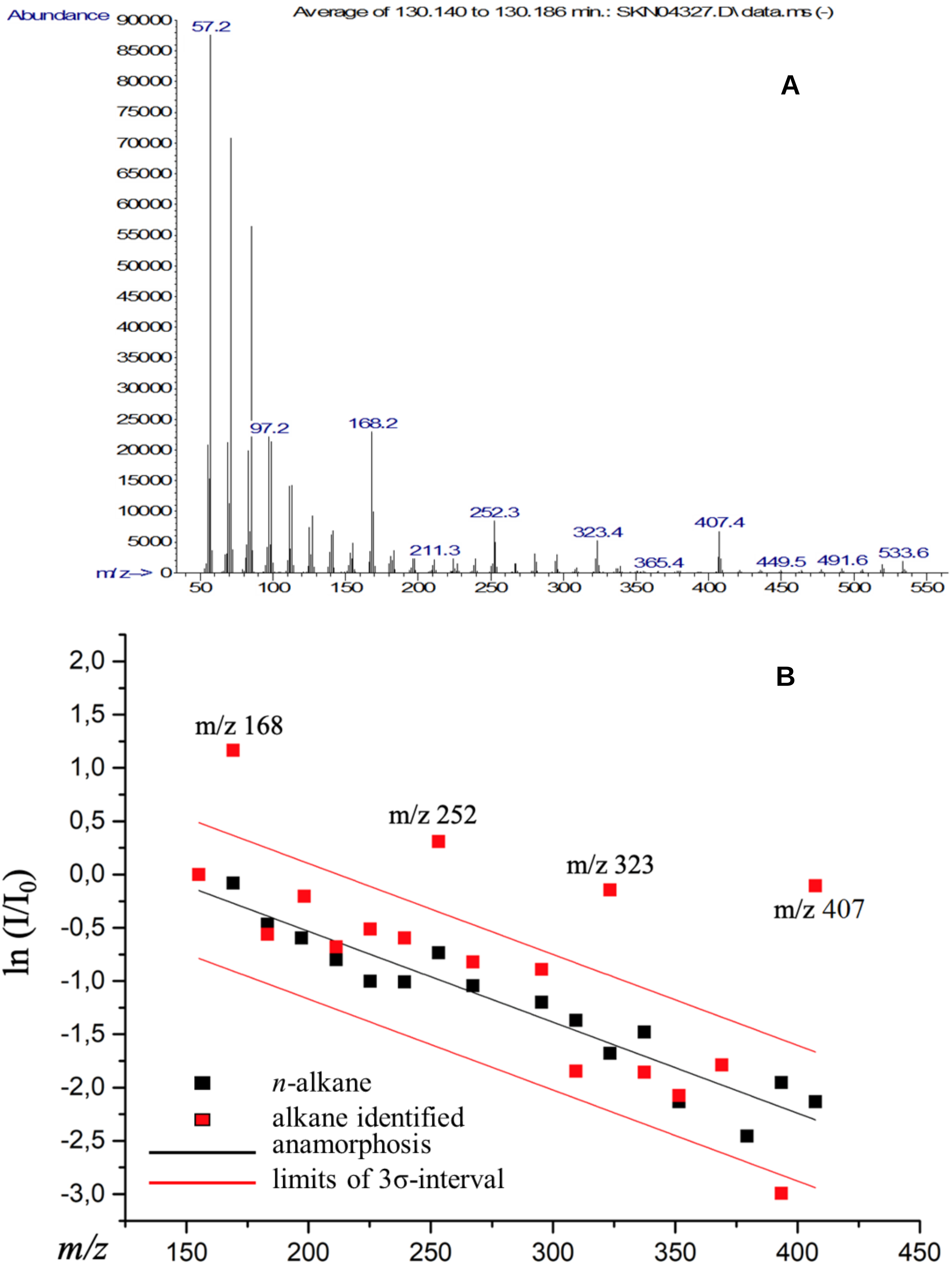

Figure S1. The mass spectrum of 11,21-dimethylheptatriacontane (A) and the scheme of detection of characteristic ions for this compound (B).

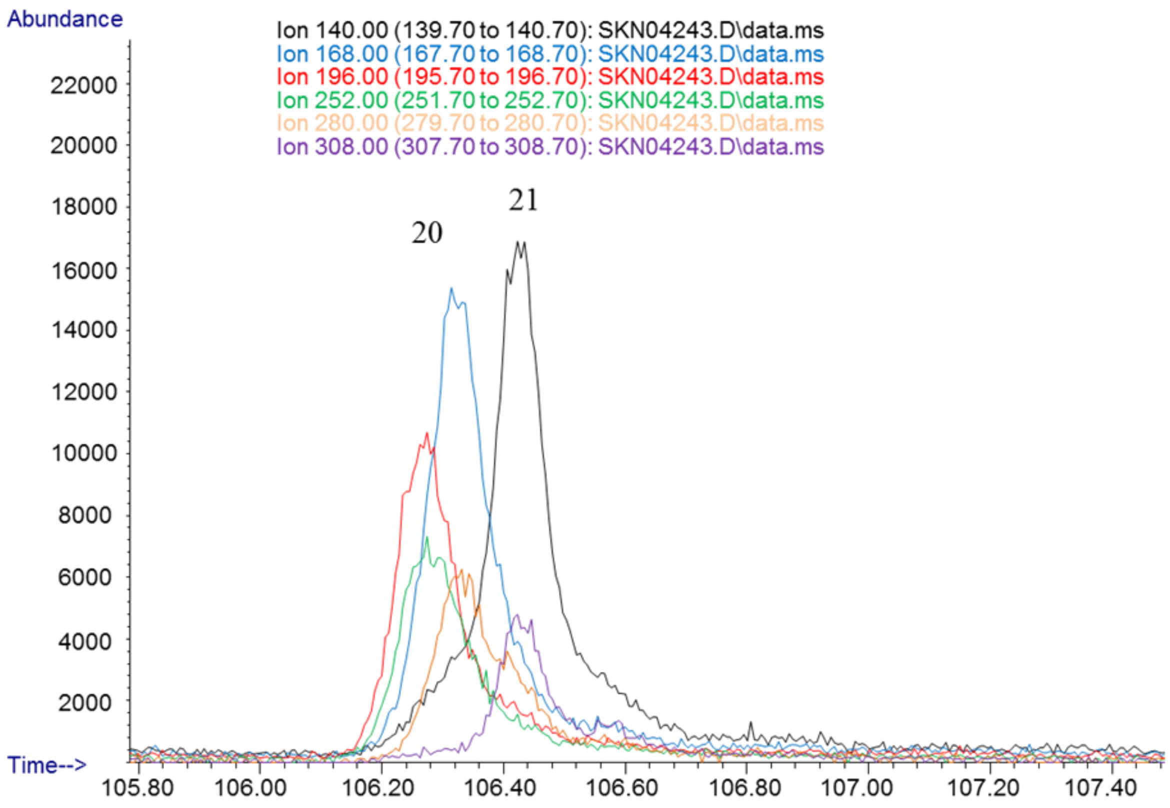

Figure S2. A fragment of a reconstructed chromatogram according to characteristic ions with *m/z* 140, 168, 196, 252, 280, and 308 in the region of peaks number 20 and 21 for *L. migratoria*.

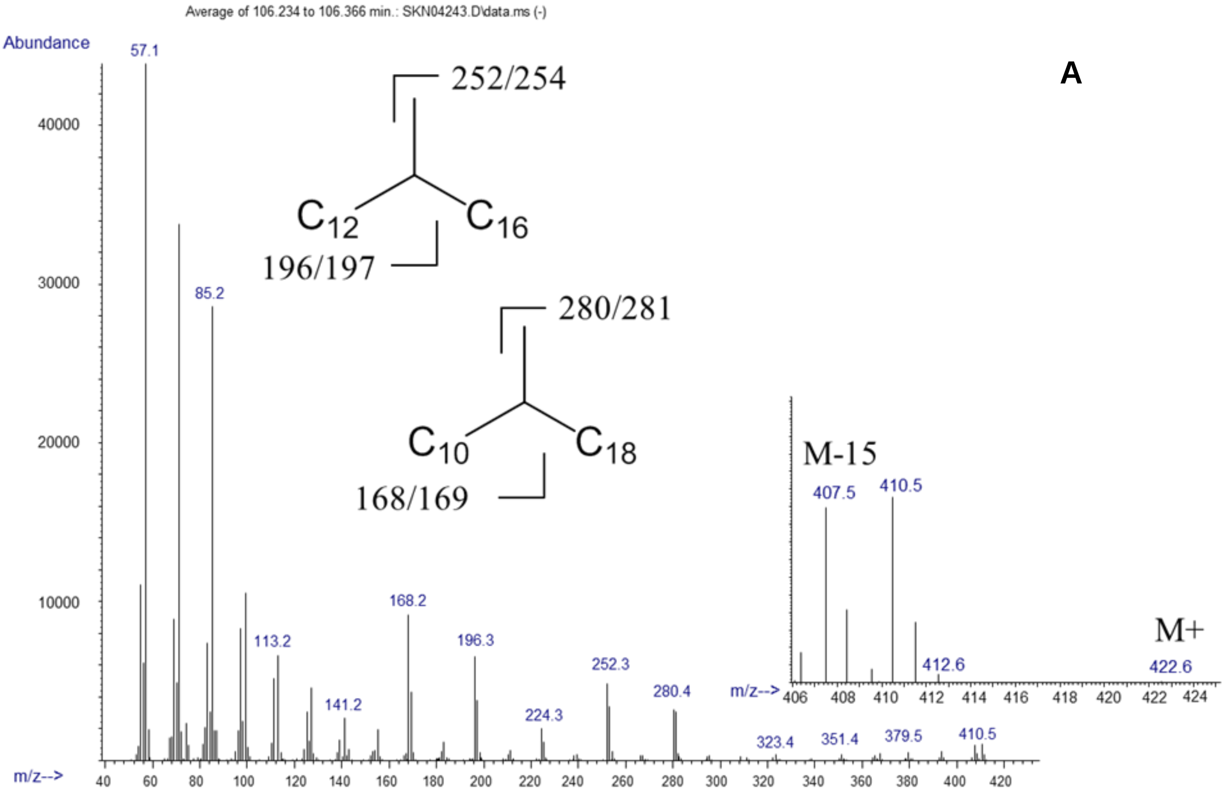

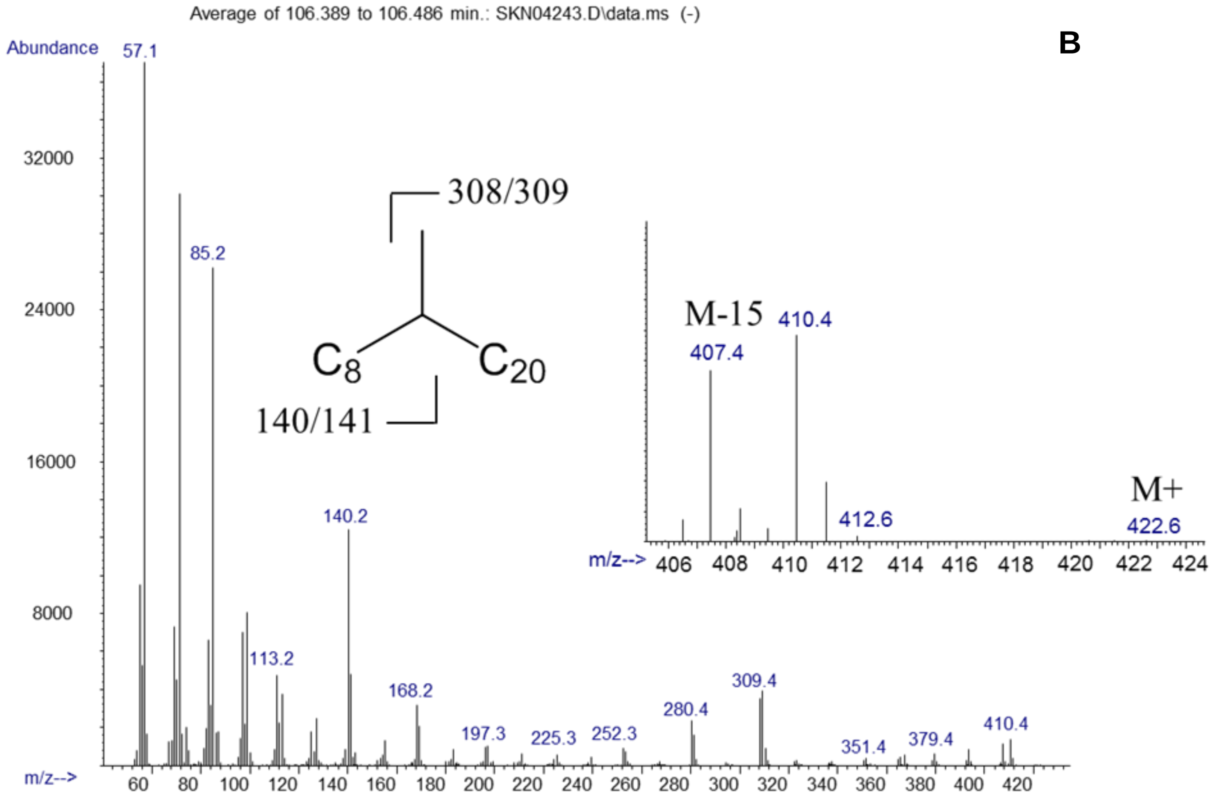

Figure S3. Mass spectra of peaks number 20 (13- and 11-methylnonacosane, panel A) and number 21 (9-methylnonacosane, panel B) for *L. migratoria* and the schemes of decay.

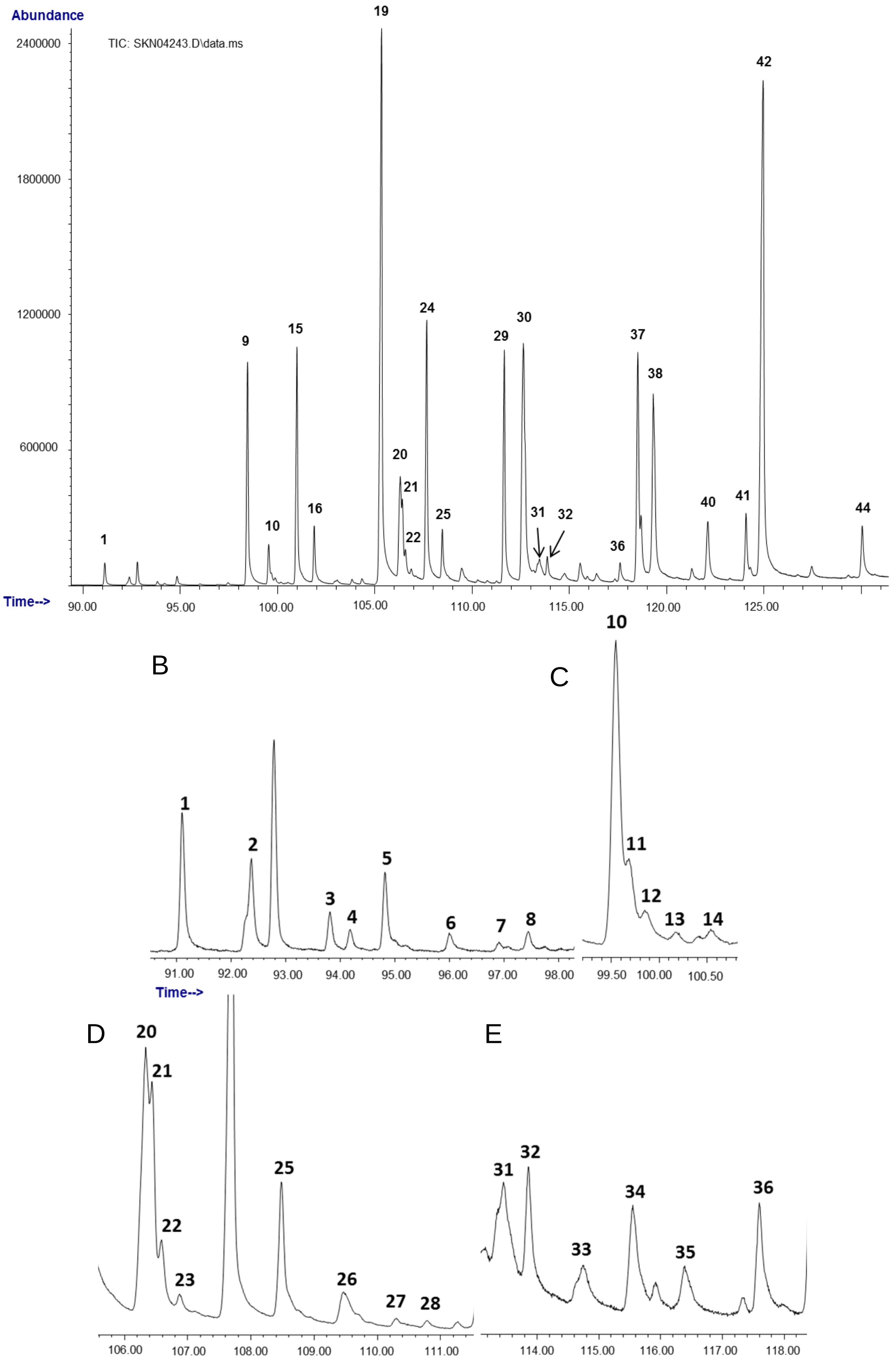

Figure S4. The chromatogram of the hydrocarbon profile of *L. migratoria* (A) and fragments of the chromatogram in the region of 90.5–98 min (B), 99–100.5 min (C) 106–111 min (D), and 113.5–118 min (E). Peak numbers correspond to Table S2.

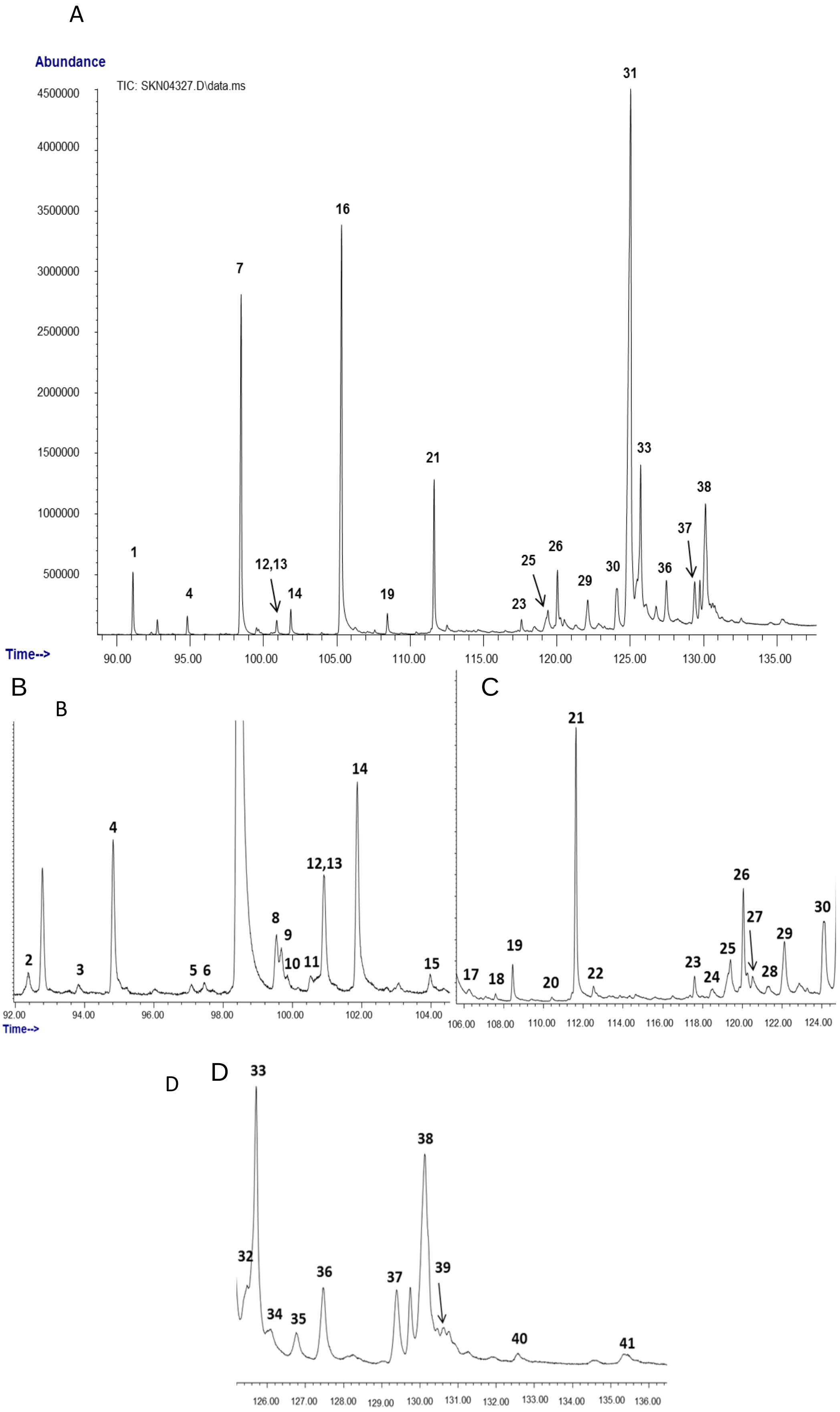

Figure S5. The chromatogram of the hydrocarbon profile of *C. italicus* (A) and fragments of the chromatogram in the region of 92–104 min (B), 106–124 min (C), and 126–136 min (D). Peak numbers correspond to Table S3.

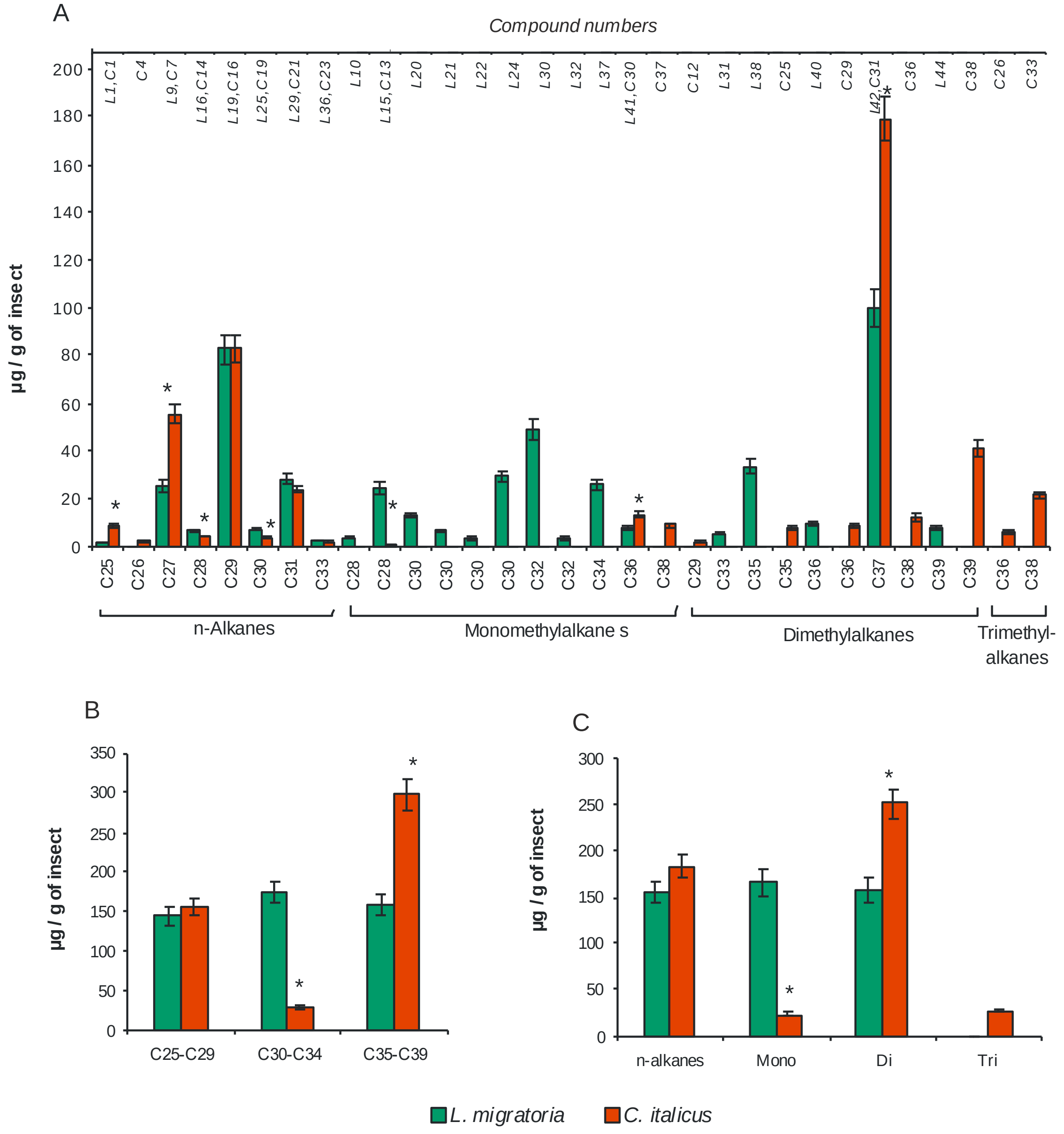

Figure S6. Amounts of major hydrocarbons in the epicuticles of *L. migratoria* and *C. italicus* larvae. **A.** Identified hydrocarbons; the lower *x*-axis denotes the total carbon number, and the upper *x*-axis indicates certain compounds for *L. migratoria* (*L*) and *C. italicus* (*C*) corresponding to Tables S2 and S3. **B**. The carbon number distribution. **C**. The distribution of methyl branches. *Significant differences between *L. migratoria* and *C. italicus* (*t* test, P < 0.05, or Mann–Whitney *U* test, P < 0.05).

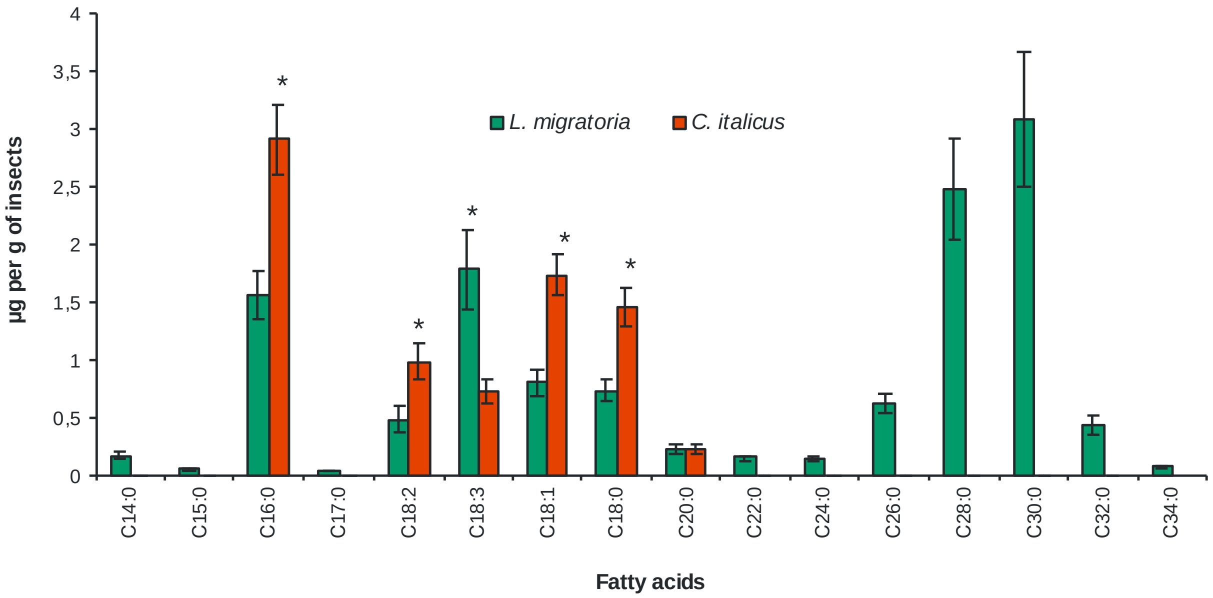

Figure S7. Amounts of fatty acids in epicuticles of *L. migratoria* and *C. italicus* larvae. *Significant differences between the species (*t* test, P < 0.05).

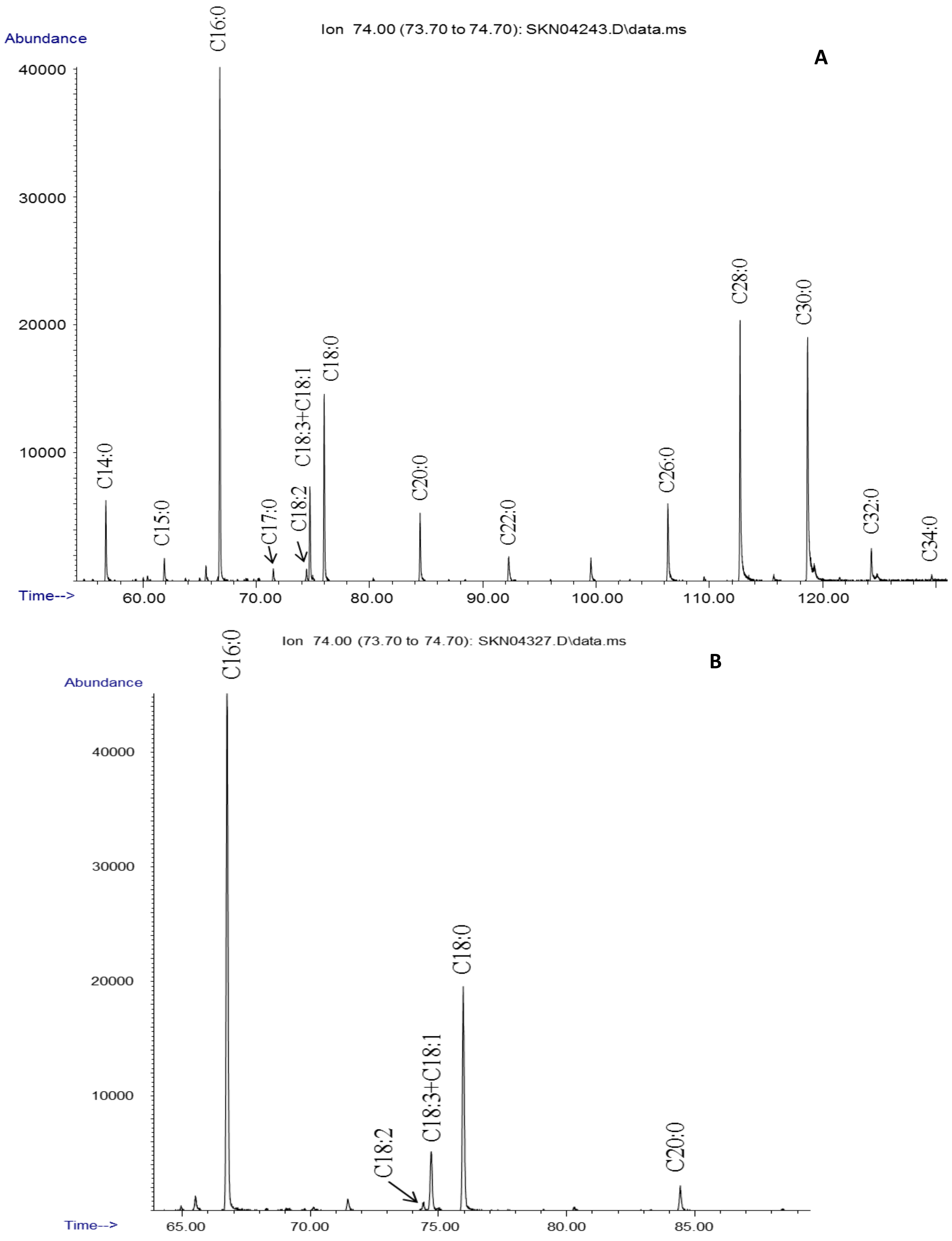

Figure S8. Fragments of a reconstructed chromatogram of fatty acids according to characteristic ions with *m/z* 74 in epicuticular extracts of *L. migratoria* (A) and *C. italicus* (B).

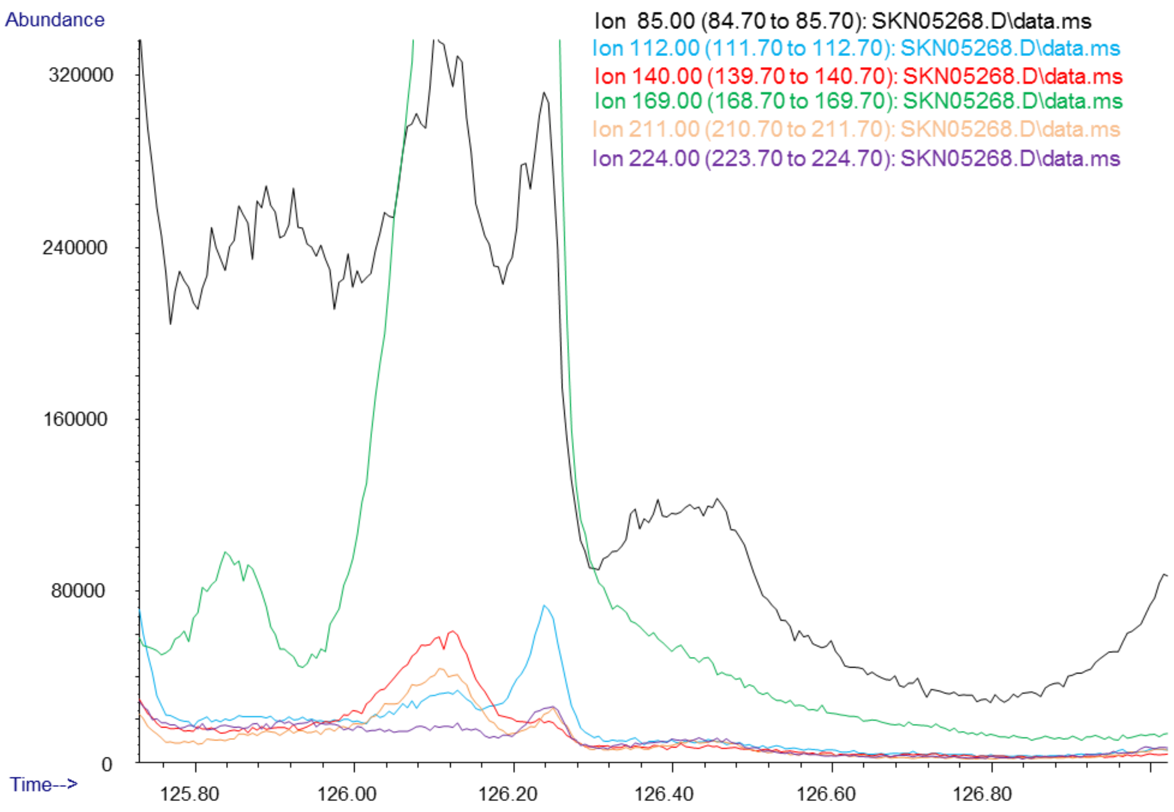

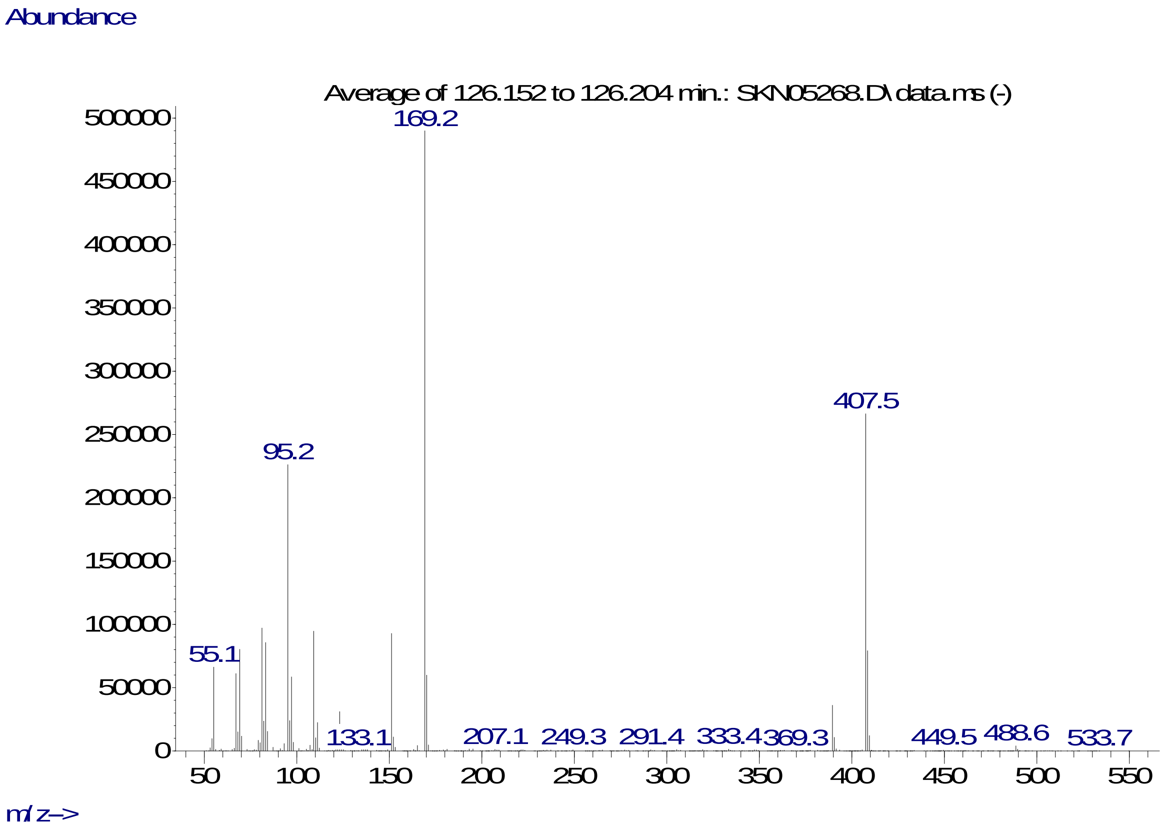

Figure S9. A fragment of a reconstructed chromatogram according to characteristic ions with *m/z* 85, 112, 140, 169, 211, and 224 in the region of hydrocarbon peaks number 33 and ketone peak number 9 for *C. italicus* (A) and a mass spectrum of this ketone (B).

Table S1. Weights of insects and epicuticular extracts.

Locusta migratoria			***Calliptamus italicus***		
Insect weight, g	Extract weight, mg	Extract weights, µg/g of insects	Insect weight, g	Extract weight, mg	Extract weights, µg/g of insects
8.118	4.85	597.4	12.48	7.94	636.2
8.882	5.4	608.0	11.735	7.3	622.1
8.67	5.1	588.2	10.762	7.03	653.2
9.242	5.6	605.9	11.888	7.76	652.8
9.622	6.11	635.0	9.766	5.7	583.7
8.648	5.25	607.1	10.06	5.53	549.7
Mean ± SD		606.9 ± 6.4	Mean ± SD		616.3 ± 16.9

Table S2. Hydrocarbons identified in the *L. migratoria* epicuticular extract.

№	LRI	Characteristic ions, *m/z*	M+, *m/z*	Carbon number	Structure	μg/ g of insect	μg / mg of extract
1	2500	-	352	C25	*n*-C25	2.05±0.24	3.4±0.4
2	2527	196/197	351 (M-15)	C26	13-methylC25	tr	
3	2566	336/337	351(M-15)	C26	3-methylC25	tr	
4	2576	84/85, 196/197, 210/211, 322/323	365 (M-15)	C27	5,13-dimethylC25	tr	
5	2600	-	366	C26	*n*-C26	tr	
6	2626	196/197, 210/211	365 (M-15)	C27	13-methylC26	tr	
7	2651	70/71, 336/337	365 (M-15)	C27	4-methylC26	tr	
8	2667	350/351	365(M-15)	C27	3-methylC26	tr	
9	2700	-	380	C27	*n*-C27	24.9±2.5	40.9±3.8
10	2726	196/197, 224/225	394	C28	13-methylC27	3.56±0.38	5.8±0.6
	2727	168/169, 252/253	394	C28	11-methylC27		
11	2730	140/141, 280/281	394	C28	9-methylC27	tr	
12	2736	112/113, 308/309	394	C28	7-methylC27	tr	
13	2744	84/85, 336/337	394	C28	5-methylC27	tr	
14	2756	168/169,196/197, 238/239, 266/267	393(M-15)	C29	11,15-dimethylC27*	tr	
15	2769	364/365	394	C28	3-methylC27	24.3±2.4	40.0±3.8
16	2800	-	394	C28	*n*-C28	6.82±0.68	11.2±1.1
17	2853	70/71, 364/365	393 (M-15)	C29	4-methylC28	tr	
18	2869	378/379	393 (M-15)	C29	3-methylC28	tr	
19	2900	-	408	C29	*n*-C29	82.6±6.4	135.8±9.5
20	2928	196/197, 252/253	422	C30	13-methylC29	12.9±1.0	21.3±1.5
	2930	168/169, 280/281	422	C30	11-methylC29		
21	2933	140/141, 308/309	422	C30	9-methylC29	6.82±0.73	11.2±1.1
22	2938	112/113, 336/337	422	C30	7-methylC29	3.33±0.40	5.5±0.6
23	2947	84/85, 364/365	407 (M-15)	C30	5-methylC29	tr	
24	2975	392/393	422	C30	3-methylC29	29.3±2.4	48.3±3.8
25	3000	-	422	C30	*n*-C30	7.08±0.56	11.6±0.9
26	3028	182/183, 280/281; 196/197, 266/267	421(M-15)	C31	13- and 12-methylC30	tr	
	3030	168/169, 294/295	421(M-15)	C31	11-methylC30	tr	
	3031	154/155, 308/309	421(M-15)	C31	10-methyl30	tr	
	3033	140/141, 322/323	421(M-15)	C31	9-methylC30	tr	
27	3056	70/71, 392/393	421(M-15)	C31	4-methylC30	tr	
28	3072	406/407	407(M-29)	C31	3-methylC30	tr	
		Cholesterol				tr	
29	3100	-	436	C31	*n*-C31	28.5±2.1	46.8±3.2
30	3131	196/197, 280/281	450	C32	13-methylC31	48.8±4.3	80.2±6.6.
	3133	168/169, 308/309	450	C32	11-methylC31		
	3135	140/141, 336/337	450	C32	9-methylC31		
31	3160	196/197, 294/295	449 (M-15)	C33	13,19-dimethylC31*	5.58±0.58	9.2±0.9
	3160	168/169, 196/197, 294/295, 322/323	449 (M-15)	C33	11,19-dimethylC31		
32	3173	420/421	450	C32	3-methylC31	3.21±0.42	5.3±0.7

33	3200	-	450	C32	*n*-C32	tr	
34	3229<br>3229<br>3230	210/211, 280/281<br>182/183, 308/309 196/197, 294/295<br>168/169, 322/323	449(M-15)<br>449(M-15)<br>449(M-15)	C33<br>C33<br>C33	14-methylC32<br>12- and 13-methylC32<br>11-methylC32	tr<br>tr<br>tr	
35	3258	182/183, 196/197, 308/309, 322/323	463 (M-15)	C34	12,20-dimethylC32	tr	
		b-sitrosterol				tr	
36	3300	-	464	C33	*n*-C33	2.61±0.29	4.3±0.5
37	3331<br>3332	196/197,308/309<br>168/169, 336/337	478<br>478	C34<br>C34	13-methylC33<br>11-methylC33	26.1±2.3	43.1±3.8
38	3359<br>3361	196/197, 322/323<br>168/169, 350/351	492<br>492	C35<br>C35	13,21-dimethylC33<br>11,23-dimethylC33*	33.7±3.4	55.3±5.4
39	3429	196/197, 322/323, 210/211, 308/309, 182/182, 336/337	477(M-15)	C35	12-, 13- and 14-methylC34	tr	
40	3459	182/193, 196/197, 336/337, 350/351	491 (M-15)	C36	12,22-dimethylC34*	9.70±0.87	16.0±1.4
41	3530	196/197, 336/337; 224/225, 308/309	506	C36	13- and 15-methylC35	7.59±0.69	12.5±1.1
42	3560<br>3563	196/197, 224/225, 322/323, 350/351<br>196/197, 350/351	520<br>520	C37<br>C37	13,21-dimethylC35<br>13,23-dimethylC35	99.9±8.1	164.3±12.4
43	3656	196/197, 211/210, 350/351, 364/365; 182/183, 224/225, 336/337, 350/351	519 (M-15)	C38	13,23 +12,22*-dimethylC36	tr	
44	3756	196/197, 224/225, 350/351, 378/379	533 (M-15)	C39	13,23-dimethylC37	7.72±0.70	12.7±1.1

* presumable structure, tr - traces

Table S3. Hydrocarbons identified in the *C. italicus* epicuticular extract.

№	LRI	Characteristic ions, *m/z*	M+, *m/z*	Carbon number	Structure	µg/ g of insect	µg / mg of extract
1	2500	-	352	C25	*n*-C25	8.85±0.75	14.4±1.3
2	2527	196/197	351(M-15)	C26	13-methylC25	tr	
3	2566	336/337	366	C26	3-methylC25	tr	
4	2600	-	366	C26	*n*-C26	2.43±0.26	4.0±0.5
5	2656	336/337	380	C27	4-methylC26	tr	
6	2667	350/351	380	C27	3-methylC26	tr	
7	2700	-	380	C27	*n*-C27	55.5±3.5	90.5±6.9
8	2726	196/197, 224/225; 168/169, 252/253	379(M-15)	C28	13- and 11-methylC27	tr	
9	2731	140/141, 280/281	379(M-15)	C28	9-methylC27	tr	
10	2736	112/113, 308/309	379(M-15)	C28	7-methylC27	tr	
11	2755	168/169,196/197, 238/239, 266/267	393(M-15)	C29	11,15-dimethylC27*	tr	
12	2767	112/113, 196/197, 238/239, 322/323	393 (M-15)	C29	7,15-dimethylC27*	2.22±0.21	3.7±0.4
13	2767	364/365	394	C28	3-methylC27	0.55±0.07	0.9±0.1
14	2800	-	394	C28	*n*-C28	4.03±0.42	6.6±0.8
15	2858	364/365	408	C29	4-methylC28	tr	
16	2900	-	408	C29	*n*-C29	82.7±5.4	134.9±10.2
17	2927	197/196, 252/253, 224/225	407(M-15)	C30	13- and 15-methylC29	tr	
	2928	168/169, 280/281	407(M-15)	C30	11-methylC29	tr	
18	2970	392/393	407(M-15)	C30	3-methylC29	tr	
19	3000	-	422	C30	*n*-C30	3.45±0.33	5.6±0.6
20	3060	392/393	421(M-15)	C31	4-methylC30	tr	
21	3100	-	436	C31	*n*-C31	23.9±1.8	39.1±3.6
22	3128	168/169, 196/197, 280/281, 308/309	435(M-15)	C32	13- and 11-methylC31	tr	
	3134	140/141, 336/337	435(M-15)	C32	9-methylC31		
23	3300	-	464	C33	*n*-C33	2.08±0.31	3.4±0.6
24	3327	196/197, 224/225, 280/281, 308/309	463(M-15)	C34	15- and 13-methylC33	tr	
	3331	168/169, 336/337	463(M-15)	C34	11-methylC33		
25	3354	196/197, 224/225, 294/295, 322/323	477 (M-15)	C35	13,19-dimethylC33	7.88±0.87	12.9±1.5
	3358	168/169, 224/225, 294/295, 350/351	477 (M-15)	C35	11,19-dimethylC33		
	3362	140/141, 224/225, 294/295, 378/379	477 (M-15)	C35	9,19-dimethylC33		
26	3384	140/141, 210/211, 224/225, 308/309, 322/323, 392/393	506	C36	9,13,19*-trimethylC33	6.03±0.50	9.8±0.9
27	3402	84/85, 224/225, 238/239, 294/295, 308/309, 448/449	506	C36	5,15,19-trimethylC33*	tr	
	3406	84/85, 182/183, 196/197, 336/337, 350/351, 448/449	506	C36	5,11,21-trimethylC33*	tr	

28	3432	224/225, 238/239, 280/281, 294/295	492	C35	15- and 16-methylC34	tr	
	3435	182/183, 196/197, 322/323, 336/337	492	C35	12- and 13-methylC34	tr	
	3437	168/169, 350/351	492	C35	11-methylC34	tr	
29	3458	168/169, 238/239, 294/295, 364/365	491 (M-15)	C36	11,15-dimethylC34*	8.48±0.77	13.9±1.4
30	3528	252/253, 280/281	506	C36	17-methylC35	13.4±1.5	21.9±2.8
	3529	224/225, 308/309	506	C36	15-methylC35		
	3530	196/197, 336/337	506	C36	13-methylC35		
	3532	168/169, 364/365	506	C36	11-methylC35		
31	3558	196/197, 252/253, 294/295, 350/351	520	C37	13,19-dimethylC35	179.0±9.4	292.0±19.1
	3561	168/169, 252/253, 294/295, 378/379	520	C37	11,19-dimethylC35		
	3564	168/169, 378/379	520	C37	11,25-dimethyl35*		
	3566	168/169, 224/225, 322/323, 378/379	520	C37	11,21-dimethylC35*		
32	3582	168/169, 196/197, 238/239, 322/323, 364/365, 392/393	519 (M-15)	C38	11,15,23-trimethylC35	tr	
33	3587	140/141, 210/211, 252/253, 308/309, 350/351, 420/421	534	C38	9,13,19-trimethylC35*	21.7±1.0	35.3±2.2
	3590	112/113, 224/225, 266/267, 294/295, 336/337, 448/449	534	C38	7,17,21-trimethylC35*		
34	3605	84/85, 210/211, 224/225, 350/351, 336/337, 476/477	534	C38	5,13,21-trimethylC35*	tr	
		84/85, 182/183, 224/225, 336/337, 378/379, 476/477	534	C38	5,11,21-trimethylC35*	tr	
		84/85, 154/155, 224/225, 336/337, 406/407, 476/477	534	C38	5,9,21-trimethylC35*	tr	
35	3630	182/183, 196/197, 224/225, 322/323, 350/351, 364/365	520	C37	12-,13- and 15-methylC36*	tr	
36	3656	168/169, 182/183, 252/253, 266/267, 294/295, 308/309, 378/379, 392/393	534	C38	12,20- and 11,19-dimethylC36	12.1±1.5	19.9±2.9
37	3727	252/253, 308/309	534	C38	17-methylC37	9.08±0.99	14.8±1.8
	3731	196/197, 364/365	534	C38	13-methylC37		
	3733	168/169, 392/393	534	C38	11-methylC37		
38	3757	196/197, 378/379	548	C39	13,25-dimethylC37	40.9±3.5	66.9±6.8
	3760	168/169, 252/253, 322/323, 406/407	548	C39	11,21-dimethylC37*		
39	3779	168/169, 238/239, 252/253, 336/337, 350/351, 420/421	547 (M-15)	C40	11,15,21-trimethylC37*	tr	

40	3854	182/183, 252/253, 280/281, 308/309, 336/337, 406/407	547 (M-15)	C40	12,20*- and 12,22*-dimethylC38	tr
41	3950	168/169, 434/435	547 (M-29)	C41	11,29-dimethylC39*	tr
	3953	168/169, 196/197, 406/407, 434/435	547 (M-29)	C41	11,27-dimethylC39*	tr
	3955	140/141, 462/463	547 (M-29)	C41	9,31-dimethylC39*	tr

*presumable structure, tr – traces

Table S4. Fatty acid methyl esters identified in the *L. migratoria* epicuticular extract.

№	LRI	M+ (*m/z*)	Structure	Name	µg / mg of extract	µg / g of insect
1	1726	242	C14:0	Methyl tetradecanoate	0.29±0.06	0.17 ±0.04
2	1827	256	C15:0	Methyl pentadecanoate	0.09 ±0.02	0.06 ±0.01
3	1927	270	C16:0	Methyl hexadecanoate	2.59 ±0.35	1.56 ±0.21
4	2025	284	C17:0	Methyl heptadecanoate	0.07 ±0.01	0.04 ±0.01
5	2091	294	C18:2	Methyl linoleate	0.81 ±0.19	0.49 ±0.11
6	2097	292	C18:3	Methyl linolenate	2.95 ±0.57	1.78 ±0.34
7	2099	296	C18:1	Methyl oleate	1.33 ±0.19	0.80 ±0.11
8	2127	298	C18:0	Methyl octadecanoate	1.21 ±0.16	0.73 ±0.09
9	2325	326	C20:0	Methyl eicosanoate	0.37 ±0.06	0.22 ±0.03
10	2524	354	C22:0	Methyl docosanoate	0.25 ±0.04	0.15 ±0.02
11	2726	382	C24:0	Methyl tetracosanoate	0.24 ±0.04	0.14 ±0.02
12	2931	410	C26:0	Methyl hexacosanoate	1.02 ±0.14	0.62 ±0.09
13	3134	438	C28:0	Methyl octacosanoate	4.09 ±0.71	2.48 ±0.43
14	3336	466	C30:0	Methyl triacontanoate	5.08 ±0.95	3.08 ±0.57
15	3539	494	C32:0	Methyl dotriacontanoate	0.73 ±0.13	0.44 ±0.08
16	3740	522	C34:0	Methyl tetratriacontanoate	0.11 ±0.01	0.07 ±0.01
Total:					21.1±2.5	12.8±1.5

Table S5. Fatty acid methyl esters identified in the *C. italicus* epicuticular extract.

№	LRI	M+ (*m/z*)	Structure	Name	µg / mg of extract	µg / g of insect
1	1927	270	C16:0	Methyl hexadecanoate	4.8±0.5	2.9 ±0.3
2	2091	294	C18:2	Methyl linoleate	1.6 ±0.2	0.98 ±0.16
3	2097	292	C18:3	Methyl linolenate	2.1± 0.4	1.31 ±0.23
4	2099	296	C18:1	Methyl oleate	2.8 ±0.3	1.74 ±0.17
5	2127	298	C18:0	Methyl octadecanoate	2.4 ±0.3	1.45 ±0.17
6	2325	326	C20:0	Methyl eicosanoate	0.36 ±0.06	0.22 ±0.04
Total:					14.1±1.7	8.6±0.9

Table S6. Ketones identified in the *C. italicus* epicuticular extract.

№	LRI	Structure	Characteristic ions	Carbon number	µg/mg of extracts	µg/g of insect
1	3180	tetratriacontan-11-one	169, 351	C34	tr*	
2	3188	tetratriacontan-9-one	141, 379	C34	tr	
3	3281	pentatriacontan-11-one	169, 365	C35	tr	
4	3285	pentatriacontan-10-one	155, 379	C35	tr	
5	3384	hexatriacontan-11-one	169, 379	C36	9.0±1.3	0.54±0.08
6	3392	hexatriacontan-9-one	141, 407	C36	tr	
7	3485	heptatriacontan-11-one	169, 393	C37	tr	
8	3488	heptatriacontan-10-one	155, 407	C37	tr	
9	3590	octatriacontan-11-one	169, 407	C38	41.8±2.7	2.6±0.15
10	3685	nonatriacontan-12-one	183, 407	C39	tr	
11	3688	nonatriacontan-11-one	169, 421	C39	tr	
12	3784	tetracontan-13-one	197, 407	C40	tr	
Total:					50.8±3.6	3.1±0.2

* traces
